# ATP released by ultrasound targeted microbubble cavitation induces vascular inflammation and improves immune checkpoint blockade efficacy

**DOI:** 10.7150/thno.105857

**Published:** 2025-04-11

**Authors:** Sepideh Jahangiri, Samuel Bourdages, Emma Skora, John Stagg, François Yu

**Affiliations:** 1 Microbubble Theranostics Laboratory, Imaging and engineering axis, CHUM Research Center, Montreal, Canada.; 2 Institut du Cancer de Montréal, Centre de Recherche du Centre Hospitalier de l'Université de Montréal, Montréal, Canada.; 3 Faculty of Medicine, Université de Montréal, Montreal, Canada.; 4 Biomedical Engineering Institute, Université de Montréal, Montreal, Canada.; 5 Faculty of Pharmacy, Université de Montréal, Montreal, Canada.; 6 Department of Radiology, Radiation Oncology and Nuclear Medicine, Faculty of Medicine, Université de Montréal, Montreal, Canada.

**Keywords:** eATP, microbubble, ultrasound targeted microbubble cavitation, vascular inflammation, immune checkpoint blockade, aPDL1

## Abstract

**Rationale:** Extracellular ATP (eATP) is a potent immune stimulant that functions as a damage-associated molecular pattern. The regulation of eATP is primarily mediated by cell surface ecto-nucleotidases (CD39 and CD73) which hydrolyze ATP into adenosine, a potent immune suppressor. CD39 and CD73 are upregulated in most cancers. Therapeutic strategies aimed at increasing ATP release in the tumor microenvironment or inhibiting adenosine activity are active areas of research in immuno-oncology. Ultrasound-Targeted Microbubble Cavitation (UTMC) is an externally applied, spatially targeted approach that has demonstrated synergy with immune checkpoint blockade (ICB) in solid tumors. However, the underlying mechanisms and optimal therapeutic combinations remain under investigation. We hypothesized that modulating purinergic signaling by UTMC could further leverage ICB efficacy.

**Methodologies:** Here, we investigated non-ablative and flow-preserving UTMC to enhance ATP release and induce inflammatory responses in a murine syngeneic colorectal tumor model (MC38) with and without CD39 inhibition. We compared two UTMC pressures (400 and 850 kPa), evaluating their impact on tumor blood flow by contrast perfusion imaging, their ability to release ATP using bioluminescence, their effect on vascular inflammation and cancer cell death through histological analysis, their synergy with aPDL1 to improve ICB efficacy, immune cell infiltration to the tumor, and immune cell drainage to the tumor-draining lymph nodes (TDLN).

**Results:** UTMC at 850 kPa and in CD39 knockout model released higher eATP concentrations, which correlated with increased vascular inflammation, enhanced cancer cell death, and reduced cancer cell proliferation. The combination of aPDL1 with UTMC and CD39 blockade significantly reduced tumor growth. This treatment also increased cytotoxic T cells (CTL), the CTL/Treg ratio, dendritic cells, and M1-prototype tumor-associated macrophages, while reducing M2-prototype macrophages within the tumor. In the TDLNs, the fully combined treatment elevated CTLs, dendritic cells, and M1-prototype macrophages, with a concurrent reduction in M2-prototype macrophages.

**Conclusion:** Our findings support that purinergic signaling can be leveraged in combination with UTMC to improve ICB therapy.

## Introduction

ATP and adenosine (Ado) are critical metabolic and immune regulators that modulate cancer immunosuppression in the tumor microenvironment (TME). Extracellular ATP (eATP) is a potent immune stimulant and functions as a Danger-Associated Molecular Pattern (DAMP) 1. In the TME, eATP induces immunogenic cell death (ICD) and secretion of immune-stimulating factors, including high-mobility group protein B1 (HMGB1), calreticulin, and type I interferons 2. eATP affects cancer cell growth, metastasis, and inflammatory response 3-5. Low eATP concentrations typically foster tumor growth and immune suppression, whereas high eATP levels activate inflammatory cells, enhancing antitumor immunity 6, 7. eATP at high concentrations activates P2X7R, which is found on macrophages, dendritic cells (DCs), granulocytes, T and B cells 6, 8-10. P2X7R activation facilitates NLRP3 inflammasome assembly and the subsequent release of IL-18 and IL-1β to recruit anti-tumor immune responses 11. Conversely, Ado, the downstream product of ATP hydrolysis, is a potent immuno-suppressor and tumor growth promoter 12. Ado ligation to its receptors, mainly A2AR, initiates immune-suppressive signaling and causes T cell exhaustion and NK cell suppression 13. The eATP/Ado balance is regulated by cell surface ecto-nucleotidases, chiefly CD39 and CD73. CD39 hydrolyzes ATP to ADP and AMP, whereas CD73 degrades AMP to Ado 12. Interestingly, CD39 and CD73 are usually overexpressed in the hypoxic TME 14. Hypoxia enhances CD39 and CD73 expression on tumor cells, immune cells (such as regulatory T cells (Treg), myeloid-derived suppressor cells (MDSCs), macrophages, DCs, and Th17), endothelial cells, fibroblasts, and mesenchymal stromal cells, leading to increase Ado production 12, 14, 15. CD73 and/or CD39 deletion help anti-tumor immune responses and improve mice survival 16, 17, suggesting their implication in tumor progression and immune suppression. Alternatively, overexpression of CD39 and CD73 is associated with a poor prognosis and tumor progression in several cancer types 5. The therapeutic strategies that increase ATP concentration within the TME or inhibit Ado activity are active research areas in immuno-oncology 12, 18, 19, including in combination with immune checkpoint blockade (ICB) 14, 18, 20.

ICB can reactivate the host's immune system to combat cancer. By inhibiting immune-suppressive pathways like PD-1/PD-L1 and CTLA-4, ICB leads to improved outcomes in various cancers, including an overall response rate of 26% in melanoma patients. However, it also typically causes immune-related side effects 5, 21, 22. Increasing the ICB dose to achieve a higher success rate is not always a reliable solution, as it can substantially amplify these side effects 23. Hence, to enhance the efficacy of immunotherapy while minimizing systemic toxicities, it is crucial to gain a deeper understanding of therapeutic resistance mechanisms, develop strategies to overcome them, and enhance the efficacy of ICBs across a wider range of cancer types and in larger patient cohorts.

Focused ultrasound (FUS) is a non-invasive focal therapeutic technique that utilizes high-frequency sound waves guided by imaging to treat deep tissue structures, which can be used for cancer ablation 24-27 and local drug delivery 28, 29 including CAR-T cell therapy 30, 31, and ICB delivery in solid tumors 5, 32-39. Interestingly, it has recently been found that ablative FUS possesses immunomodulation effects, turning a cold TME into a hot TME, hence making FUS a potential enhancer of immunotherapy 5, 25. Several studies have demonstrated that FUS can induce ICD and enhance antigen presentation, transforming an immunosuppressive TME into an inflamed immune-active one 25, 34. However, the underlying mechanisms and optimal therapeutic combinations remain under investigation. FUS is also typically ablative, which may hinder the subsequent access of drugs and immune cells to the ablated area.

Ultrasound-Targeted Microbubble Cavitation (UTMC) is a FUS modality that utilizes low-intensity pulsed ultrasound at low to moderate pressure levels, typically between 0.1 and 4 MPa, combined with the systemic administration of microbubbles (MB) 5. Under US stimulation, MBs undergo stable and inertial cavitation, producing shear stress on nearby cell membranes and vessel boundaries, which can lead to the breakdown of tight junctions between vascular endothelial cells, compromising vessel integrity 40, 41. Non-ablative UTMC treatment causes mechanical perturbation that enhances antibody delivery in several cancers 5, 36. A promising example of UTMC application for enhanced antibody delivery was recently reported by Meng et al. (2021) in a single-armed, open-labelled clinical study (NCT03714243) using an MRI-guided focused ultrasound system for enhancing Trastuzumab delivery in four patients with progressive intracranial HER2-positive brain metastases 42. UTMC treatment can enhance tumor perfusion, relieve hypoxia, and thus improve radiotherapy efficacy in a rodent model 40. Recently, non-ablative UTMC was shown to increase DAMPs such as HMGB1, HSP60, HSP70, and calreticulin in tumor tissue, stimulating adaptive immune responses 33, 40, 43. Interestingly, UTMC was also reported to release ATP in mice muscle 44 and tumors 45. Therefore, these reports highlight the importance of non-ablative UTMC and its potential to improve adjuvant cancer therapy.

In this study, we are interested in investigating whether non-ablative, flow-preserving UTMC, could induce antitumor inflammatory responses in MC38 tumors with and without CD39 inhibition. We hypothesized that this mechanism is ATP-dependent and involves vascular inflammation. To the best of our knowledge, the importance of ATP release by UTMC in the context of ICB had not been studied, nor has the role of ATP in vascular inflammation post-UTMC. To determine which US pressure would be more effective at inducing anti-tumor inflammatory responses, US pressures of 400 and 850 kPa were studied. Therefore, our objectives were: 1) to determine if UTMC enhances eATP with CD39 inhibition, 2) to investigate whether enhanced ATP with CD39 inhibition induces vascular inflammation, 3) to assess if enhanced ATP causes cancer cell death, 4) to assess if CD39 inhibition can enhance UTMC + ICB efficacy, and 5) to determine if the immune cell responses in TME are shifted toward an inflamed TME.

## Material and Methods

### Cells and animals

The mouse colorectal carcinoma cell line (MC38), kindly provided by Dr. Stagg (CRCHUM), was cultured in high-glucose RPMI 1640 (Wisent) supplemented with L-glutamine, 1.5 g/L sodium bicarbonate, sodium pyruvate, HEPES, 10% fetal bovine serum (Wisent), and 100 U/ml penicillin/streptomycin (Wisent) under 5% CO_2_ at 37 °C. Eight to ten-week-old female C57BL/6 wild-type mice (WT) were purchased from Charles River (Canada). CD39 knockout mice (CD39KO) with a C57BL/6 background, kindly provided by Dr. Stagg, were bred and maintained at the Centre de Recherche du Centre Hospitalier de l'Université de Montréal. 2.5×10^5^ MC38 cells in a mixture (1:1) with Matrigel (Corning, Tewksbury, MA) were subcutaneously inoculated into the posterior upper flank area of WT or CD39KO female mice (100 µl/side). Tumor size was measured using calipers every 2-3 days, and the volume was calculated as (length × width × depth/2). All animal studies were approved by the Institutional Animal Protection Committee of the University of Montreal Hospital Research Center (ethical approval number #2I23011FYs), which holds a Certificate of Good Animal Practice from the Canadian Council on Animal Care.

### Ultrasound image guided therapy

A 7 MHz central frequency ultrasonic imaging probe (15L8 probe, Sequoia, Siemens) was placed horizontally aiming at the tumor, allowing contrast-enhanced ultrasound (CEUS) imaging. An MB suspension (Definity®, Lantheus) was slowly infused (4-6 µl/min) through the tail vein during the treatment and for real-time CEUS imaging. Burst replenishment cineloops were recorded (CPS7 mode; mechanical index (MI) = 0.2, Sequoia, Siemens) before, during, and after treatment. Cineloops were analysed offline by fitting a mono-exponential function (video intensity, 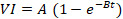) to compute the reperfusion rate AB (dB/s) 40, using a custom MATLAB program (MATLAB, version 9.8.0 (R2020a); Natick, Massachusetts), as described elsewhere 40. In brief, the kinetics of MB perfusion were quantified by measuring AB parameters both pre-treatment and three minutes after treatment. The steady-state post-treatment perfusion AB (dB/s) values were averaged and normalized by muscle MB perfusion (untreated area) in the pre-treatment clip to compensate for changes in MB concentration between cineloop recordings. The therapeutic transducer (1 MHz, 0.5 inches, A303S, Olympus) was calibrated using a membrane hydrophone (ONDA HMB-0200 S/N:1423) in a degassed water tank. For tumor treatment, the single-element unfocused transducer was positioned directly over the tumor using coupling gel and perpendicularly to the imaging plane. Ultrasound pressure for efficacy studies was set at either 400 kPa or 850 kPa corresponding to MI of 0.4 and 0.85, resp. Trains of 5000 cycles were generated by a function generator (Model 33500B Trueform Waveform Generator, Keysight, Santa Rosa, CA), amplified (Model 1040, ENI, Rochester, NY, USA), and delivered every 5 seconds for 10 minutes to the tumor site.

### Treatment protocols in acute studies

**Figure 1:**
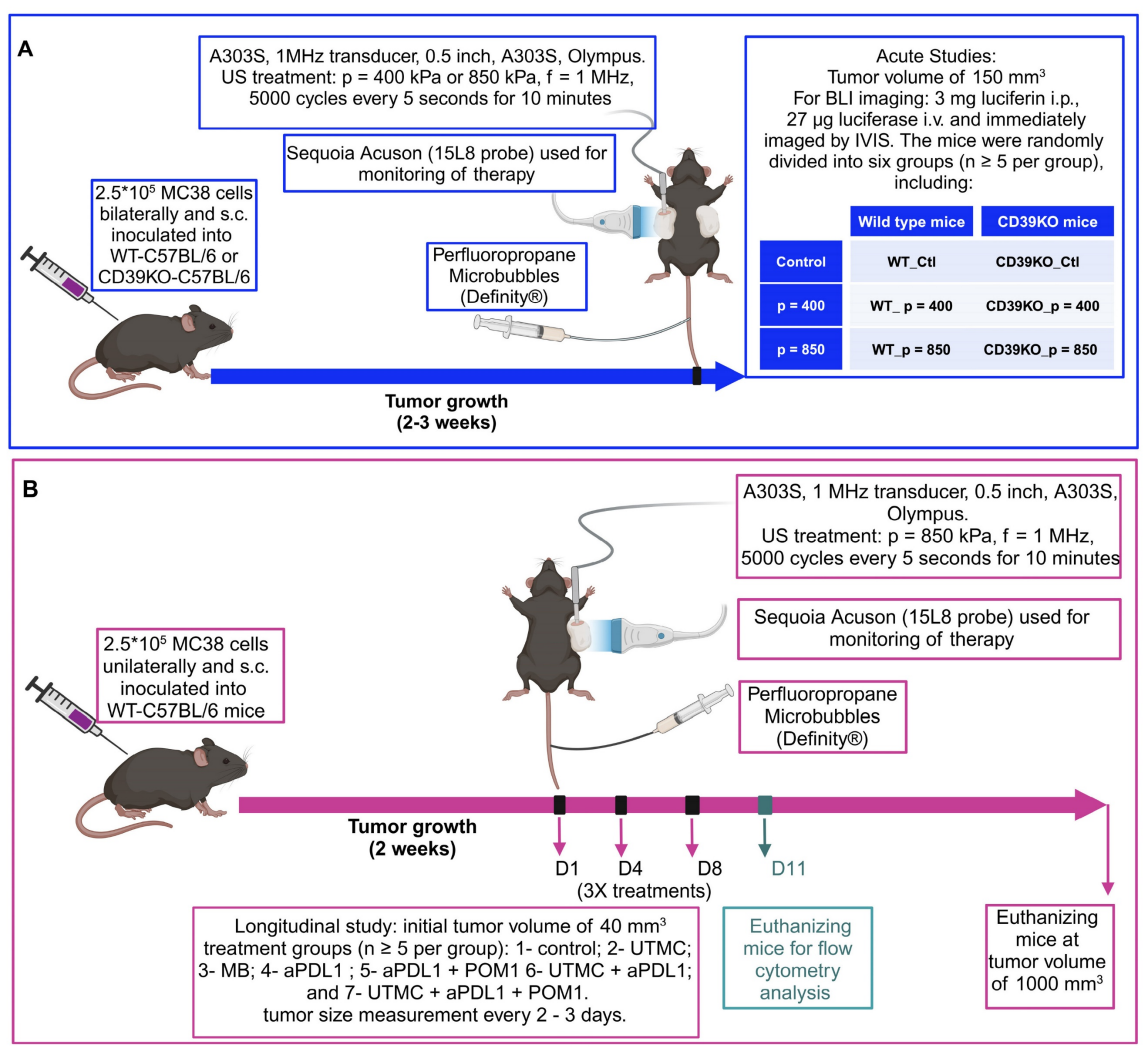
** Treatment protocols for (A) acute and (B) longitudinal studies.** 2.5 x 10^5^ MC38 cells were injected s.c. (A) bi-laterally or (B) unilaterally into flanks of wild-type or CD39KO mice. (A) At a tumor volume of 150 mm^3^, mice were treated and imaged by IVIS (at 2 h) and histological analysis (at 24 h); (B) At a tumor volume of 40 mm^3^, mice received three treatments of UTMC + aPDL1 (100 µg i.p.) + POM1 (250 µg i.p.). MBs were infused via a tail vein catheter during UTMC treatment. CEUS was used to guide the therapeutic probe placement and burst replenishment imaging was performed to assess MB perfusion before and after UTMC. Tumor size was measured longitudinally. To study immune responses, mice were euthanized three days after treatment #3, at which time (D11) tumor samples and TDLNs were collected. aPDL1: anti-programmed cell death ligand-1; BLI: bioluminescence imaging; CD39KO-C57BL/6: CD39 knockout mice; Contrast enhanced ultrasound (CEUS); i.v.: intravenous injection; POM-1: Sodium polyoxotungstate-1; s.c.: subcutaneous; TDLN: tumor-draining lymph nodes US: ultrasound; WT-C57BL/6; wild-type mice.

Before treatment, the mice were anesthetized with ketamine (50 mg/kg) + Dexmedetomidine (0.5 mg/kg) and isoflurane 0.5% (if needed for maintenance). As shown in **Figure 1A**, when the tumor volume reached 150 mm^3^, the mice were randomly divided into six groups (n ≥ 5 per group), including: 1- control wild type (WT_Ctl); 2- wild type treated with p = 400 kPa (WT_p = 400); 3- wild type treated with p = 850 kPa (WT_p = 850); 4- control CD39KO (CD39KO_Ctl); 5- CD39KO treated with p = 400 kPa (CD39KO_p = 400); 6- CD39KO treated with p = 850 kPa (CD39KO_p = 850). For ATP quantification, mice were immediately transferred to the IVIS chamber to measure the bioluminescent signal. These mice were then sacrificed 2 h post UTMC to quantify vasodilation on H&E slides. For histopathological investigation (CC3, ki67, CD3, ICAM, VCAM1, CD31), another group of mice were humanely euthanized 24 h after UTMC by cervical dislocation. The tumor samples were then collected, formalin-fixed, and embedded in paraffin, or were frozen in O.C.T. mounting medium (Sakura Tissue-Tek, Torrance, CA) for further analysis.

### Treatment protocols in longitudinal studies

Before treatment, the tumor-bearing mice were anesthetized with Ketamine (50 mg/kg) + Dexmedetomidine (0.5 mg/kg) or with isoflurane 1.5%. As shown in** Figure 1B**, when the tumor volume reached 40 mm^3^, the mice were randomly divided into different treatment groups (n ≥ 5 per group), including: 1- control; 2- UTMC; 3- MB; 4- aPDL1 (100 µg); 5- aPDL1 (100 µg) + POM-1 (250 µg); 6- UTMC + aPDL1 (100 µg); and 6- UTMC + aPDL1 (100 µg) + POM-1 (250 µg). The role of ATP was investigated by pharmacological CD39 inhibition (POM-1) or using a CD39 knockout mouse model (CD39KO). aPDL1 and POM-1 were administered through the intraperitoneal (i.p.) route 30 minutes before UTMC treatment. Treatments were given three times with a 2-3 day interval. Tumor volume (0.5 × Length × Width × Depth) was measured using a digital caliper daily. Mice were sacrificed if the tumor reached the end-point cut-off volume of 1000 mm^3^. Tumor collection was the same as described for acute studies. Immune cell infiltration into the tumor and immune cell trafficking to the tumor-draining lymph nodes (TDLNs) were assessed in the same longitudinal model. However, in this case, the mice were euthanized on day 11, three days after their third treatment.

### Optical imaging of eATP following UTMC

To examine changes in the level of eATP following UTMC treatment in WT or CD39KO mice, 3 mg XenoLight D-Luciferin (#122796, Perkin Elmer, Waltham, MA) was administered i.p., and 27 µg Luciferase (Promega, Canada) was administered i.v. To quantify ATP release, both tumors (UTMC-treated and the untreated (control)) were imaged for bioluminescent signal (BLI) using an IVIS Spectrum Imaging System (PerkinElmer, Waltham, MA), and the total flux (photon/second) was calculated using Living Image® software analysis (PerkinElmer, Waltham, MA).

### Histology

Paraffin-embedded specimens were cut into 5 µm sections and used for histological staining with H&E or IHC, and IF staining, using the BenchMark XT automated stainer (Ventana Medical System Inc; Tucson, AZ). Antigen retrieval was carried out with Cell Conditioning 1 (Ventana Medical System Inc; #950-123) for 90 minutes for all primary antibodies. Cell proliferation and cell apoptosis were studied through IHC staining using antibodies against Ki-67 (Cell Signaling #9449, 1:500 dilution) and CC3 (Cleaved Caspase-3; Cell Signaling #9661, 1:100 dilution), resp. IHC sections were scanned using the Leica Aperio VERSA 200 scanner (Leica Biosystems), and the level of expression of each marker was analyzed through VisiomorphDP software (VisioPharm, Hørsholm, Denmark). Normalized expression was defined as the extent area of each marker divided by the tissue area. Consecutive sections were also used for IF staining against the vascular inflammatory markers VCAM-1 and ICAM-1 within CD31-positive endothelial cells. To quantify vascular inflammation, the vasculature was first detected using CD31 signal in non-necrotic regions and marked as CD31 ROI. Then, the surface area of ICAM-1 or VCAM-1 within CD31 ROI was quantified and normalized by CD31 surface area. IF staining was performed with a cocktail of primary antibodies, including rat anti-VCAM-1 (Invitrogen, #13-1061-82, 1:100 dilution), mouse anti-ICAM-1 (Invitrogen, # MA5407, 1:500 dilution), and rabbit anti-CD31 (Abcam, ab28364, 1:25 dilution). A mixture of secondary antibodies, including goat polyclonal anti-rabbit-AF647 (Invitrogen, A-21422, 1:125 dilution), goat anti-mouse-AF555 (Invitrogen A-21244, 1:1500 dilution), and goat anti-rat-AF488 (Invitrogen, 1:100 dilution), was added and incubated for 1 hour at room temperature. Sections were then washed three times for 5 minutes each with PBS, mounted with ProLong Gold Antifade with DAPI (ThermoFisher, P36931), and sealed. IF slides were scanned with an Olympus Optical microscope BX61VSF 241 (Olympus, Shinjuku, Tokyo, Japan) using a 20X 0.75NA objective and a resolution of 0.3225 μm (Olympus Canada Inc., Richmond Hill, ON, Canada), linked to the OlyVIA® image 243 viewer software (Olympus, xvViewer.exe) and analyzed using Visiomorph software.

### Flow cytometry analysis

Tumor samples and TDLNs were collected three days after the third treatment (**Figure 1B**, on D11), minced and digested in tumor dissociating media (RPMI1640, 20ug/ml DNAse I (Roche), and 1mg/ml Collagenase type IV (Worthington Biochemical)) for 1h at 37°C. The cell suspension was then passed through a 70 µm cell strainer (Zellsieb) and washed twice in FACS buffer (PBS 1X, 2% FBS (fetal bovine serum), and 2.5 mM EDTA). Anti-CD16/32 mAb (Cytek, clone 2.4G2) was used to block Fc receptors. For extracellular marker staining, single-cell suspensions were incubated with a cocktail of viability dye (ef506, Thermo Fisher Scientific; cat.: 65-0866-14) and fluorescence-conjugated antibodies (1:200 BUV737 mouse anti-mouse CD45, 1:500 BV786 hamster anti-mouse CD3e, 1:1000 AF700 rat anti-mouse CD4, 1:200 Pacific Blue rat anti-mouse CD8a, 1:500 BUV395 rat anti-mouse CD11b, 1:50 APC hamster anti-mouse CD11c, 1:200 PE rat anti-mouse F4/80, 1:1000 BV650 rat anti-mouse CD86, and 1:200 PE-Cy7 rat anti mouse CD206) for 30 minutes on ice.

Next, for intracellular FoxP3 staining, single-cell suspensions were first fixed and permeabilized using fixation/permeabilization buffer (Invitrogen, #00-5523-00) for 30 minutes on ice, washed with permeabilization buffer (Invitrogen, #00-5523-00), and then stained with 1:200 AF488 rat anti-mouse FoxP3. Flow cytometry was conducted on an LSRFortessa^TM^ (BD Bioscience, San Jose, CA, USA) and analyzed using FlowJo software (version 10.8.0).

### Statistical analysis

Data were first tested for normality using the Shapiro Wilk test. If the data were normally distributed, parametric tests were used. Perfusion rates (AB) were analyzed using paired t-tests in longitudinal and acute studies. For comparing differences between multiple groups, two-way ANOVA followed by Tukey's multiple comparisons test was used. For non-normal data set (supplementary results), group differences were compared using the Mann-Whitney test. Mice survival was analyzed using the Log-rank Mantel-Cox test. All data are presented as mean ± SEM, and statistical significance was set at p < 0.05. All statistical analyses were performed using GraphPad Prism version 10.0 (San Diego, California, USA, www.graphpad.com).

## Results

### Tumor perfusion was maintained following UTMC

**Figure 2:**
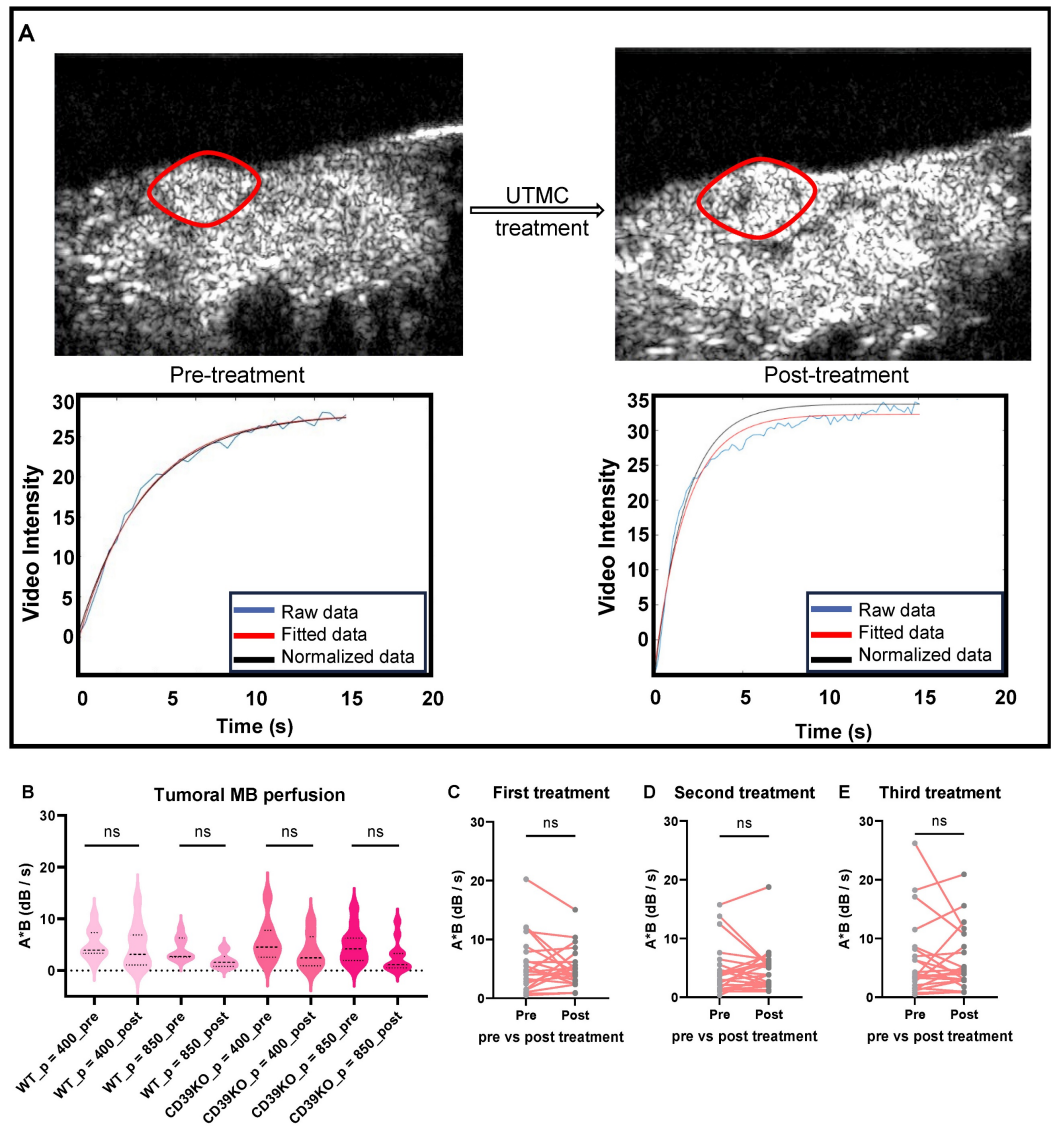
**Tumor perfusion following UTMC treatment. A)** The tumor area is highlighted with a red line. An example of burst replenishment imaging is provided to display MB perfusion before starting UTMC treatment and 5 minutes after UTMC. MB replenishment was quantified offline to derive blood volume (A) and MB perfusion (A × B) by fitting video intensity with an exponential function 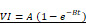, where A is the plateau video intensity [dB] (maximum MB concentration) and B is the rate constant [s^-1^ ] (blue line: raw data, red line: fitted data, black line: normalized data based on MB concentration in non-treated region (muscle) of pre-treatment video). **B)** In acute studies, MB perfusion was similar in tumors pre- vs. post-treatment, therefore UTMC was not ablative (n > 5 per group, p > 0.05 in each set of comparison, paired t-test). **C-E)** In longitudinal studies, MB perfusion did not change before and after first, second or third UTMC treatments (p = 0.45, p = 0.91, and p = 0.78, resp., paired t-test). MB: microbubble; UTMC: ultrasound targeted microbubble cavitation; VI: video intensity.

CEUS imaging was used to analyze the tumor blood perfusion before and after treatment (**Figure 2A**). The MB perfusion was quantified in both pre- and post-treatment videos using MATLAB. Comparing pre- versus post-treatment videos revealed that UTMC treatments at pressures of either 400 (p = 0.81 in WT and p = 0.19 in CD39KO mice) or 850 kPa (p = 0.33 in WT and p = 0.08 in CD39KO mice) did not shut down the tumor perfusion (**Figure 2B-C**). Additionally, MB perfusion was analysed pre- and post-UTMC on each treatment day, confirming the non-ablative treatment at the studied timepoints (**Figure 2C-E**). Hence, molecular and pathological modifications in TME were explored following non-ablative UTMC in our next steps.

### UTMC induces vasodilation in WT and CD39KO mice

**Figure 3:**
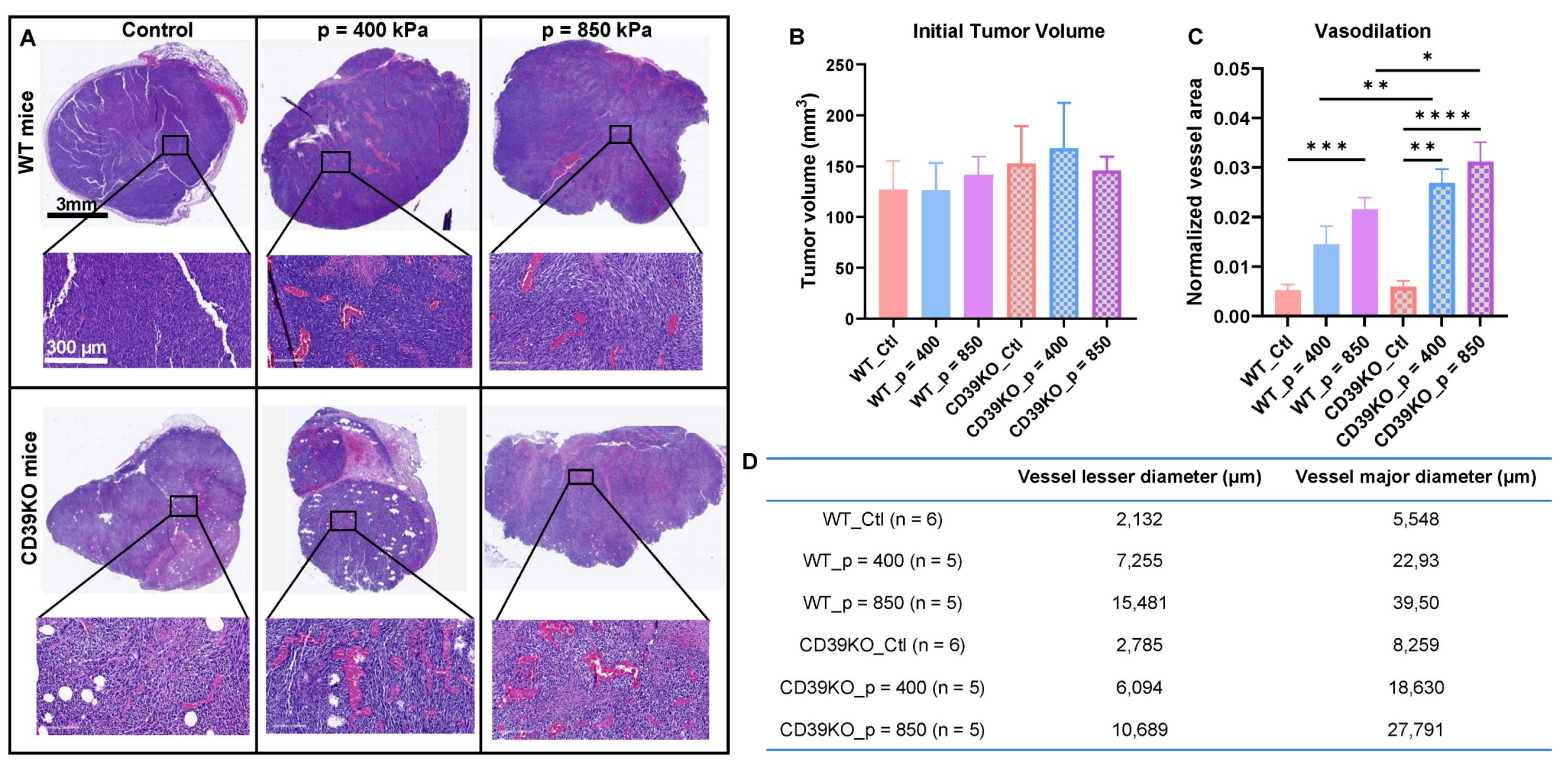
** Vasodilation following UTMC treatment. A)** Typical H&E staining on tumor samples collected 2 h after UTMC treatment. To better visualize vessel dilation, a section of vascularized section of tumor tissue is magnified on the bottom. **B)** Tumor volume in all the groups was similar (n > 5 per group). **C)** Normalized vessel area (vessel area/tissue area) in non-necrotic tissue significantly increased in WT_p = 850 compared to WT_Ctl group (p = 0.0003). In CD39KO group, the normalized vessel area was increased in CD39KO_p400 (p = 0.001) and CD39KO_p = 850 (p < 0.0001) compared to CD39KO_Ctl. Normalized vessel area was significantly increased in CD39KO versus WT mice following US pressure of 400 kPa (p = 0.006) and 850 kPa (p = 0.005). **D)** Mean of minimum and maximum vessel diameters (µm) are shown for each group. CD39KO: CD39 knock-out mice; CD39KO_Ctl: No US control group of CD39KO mice; eATP: extracellular ATP; US: ultrasound; UTMC: ultrasound targeted microbubble cavitation; WT_Ctl; No US control group of wild-type mice.

To further analyze UTMC effect on perfusion and inducing vasodilation, tumor samples were collected 2 h post-UTMC and sequential slides were stained by H&E and CD31 marker. Histological examination revealed a notable vasodilation in all UTMC-treated samples, which was absent in control slides. Enlarged blood vessels, with extensive accumulation of red blood cells in the vessel lumens, were consistently observed (**Figure 3A**). This observation was further confirmed by independent pathologists who were blinded to the mice groupings and quantified offline. Initial tumor volume was not significantly different between any of the groups (**Figure 3B**). **Figure 3C** and **D** show that normalized vessel area was increased following UTMC in WT_p = 850 compared to WT_Ctl group (p = 0.0003). In CD39KO group, the normalized vessel area was increased in CD39KO_p = 400 (p = 0.001) and CD39KO_p = 850 (p < 0.0001) compared to CD39KO_Ctl. Normalized vessel area was significantly increased in CD39KO versus WT mice following US pressure of 400 kPa (p = 0.006) and 850 kPa (p = 0.005).

### UTMC increases eATP in MC38 tumor of WT and CD39KO mice

**Figure 4:**
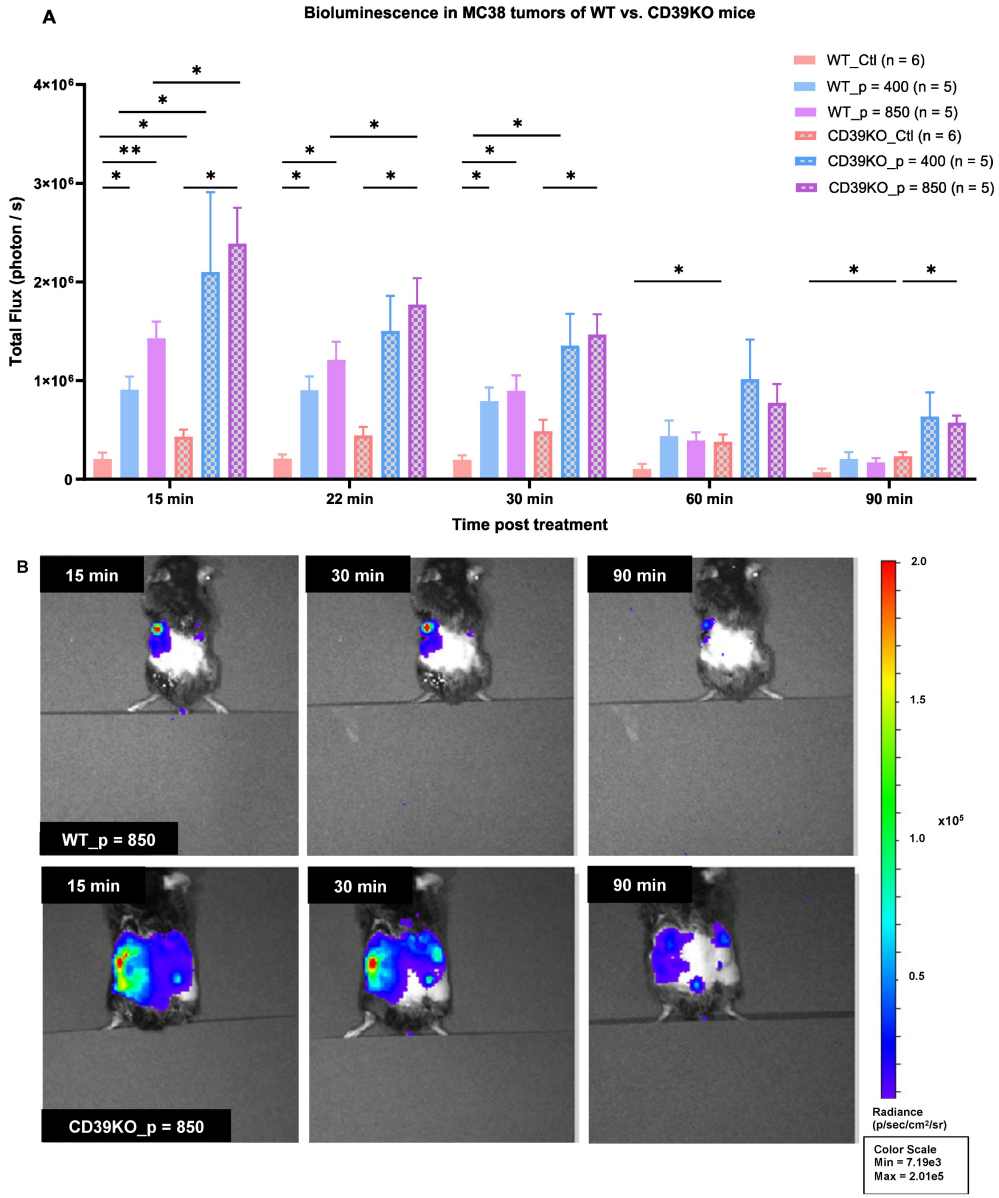
** eATP levels in MC38 tumor in WT and CD39KO mice measured by bioluminescence. A)** The baseline eATP signal in untreated control tumors was higher in CD39KO vs WT mice (p = 0.04, two-way ANOVA). UTMC treatment released more ATP in CD39KO than in WT mice (p = 0.002, two-way ANOVA). This difference was significant at both UTMC pressures at 15 min (p = 0.04 and 0.03, resp.). The increased eATP level was maintained longer in CD39KO_p = 850 until 90 min post-UTMC (p = 0.03). In the WT group, UTMC at 400 kPa and 850 kPa yielded a higher eATP signal compared to WT_Ctl until 30 minutes post-UTMC (p = 0.017 and 0.02). CD39KO_p = 850 had a higher eATP signal compared to CD39KO_Ctl up to 90 minutes post-UTMC (p = 0.011). **B)** Typical examples of BLI imaging representing local eATP released following UTMC treatment with 850 kPa in WT and CD39KO mice at 15, 30 and 90 minutes; Only left tumors received UTMC. BLI: bioluminescent imaging. CD39KO: CD39 knock-out mice; CD39KO_Ctl: No US control group in CD39KO mice; eATP: extracellular ATP; US: ultrasound; UTMC: ultrasound targeted microbubble cavitation; WT_Ctl; No US control group in wild-type mice.

MB cavitation caused by UTMC has been shown to promote ATP release in mice muscle 44 and in tumors 45, but the effect of CD39 inhibition on ATP release was never studied. UTMC-treated and control tumors were imaged for bioluminescence. Our results showed that the baseline BLI signal is significantly higher in CD39KO than WT mice (p = 0.04,** Figure 4A**). The total flux (photon/sec) was also higher for the treated side in all the mice. ATP release was significantly higher in CD39KO compared to WT mice at 15 minutes post-UTMC at US pressures of 400 and 850 kPa (p = 0.02). At 22 and 30 minutes post-UTMC, the eATP levels were higher in CD39KO vs. WT only when treated at p = 850 kPa (p = 0.03 and 0.01, resp.). In WT mice, UTMC treatment at both US pressures enhanced ATP release compared to untreated controls up to 30 minutes post-UTMC. In CD39KO mice, UTMC enhanced ATP release at a pressure of 850 kPa compared to untreated control up to 90 minutes post-UTMC treatment (**Figure 4B**).

### UTMC induces tumoral vascular inflammation in CD39KO mice but not in WT mice

**Figure 5:**
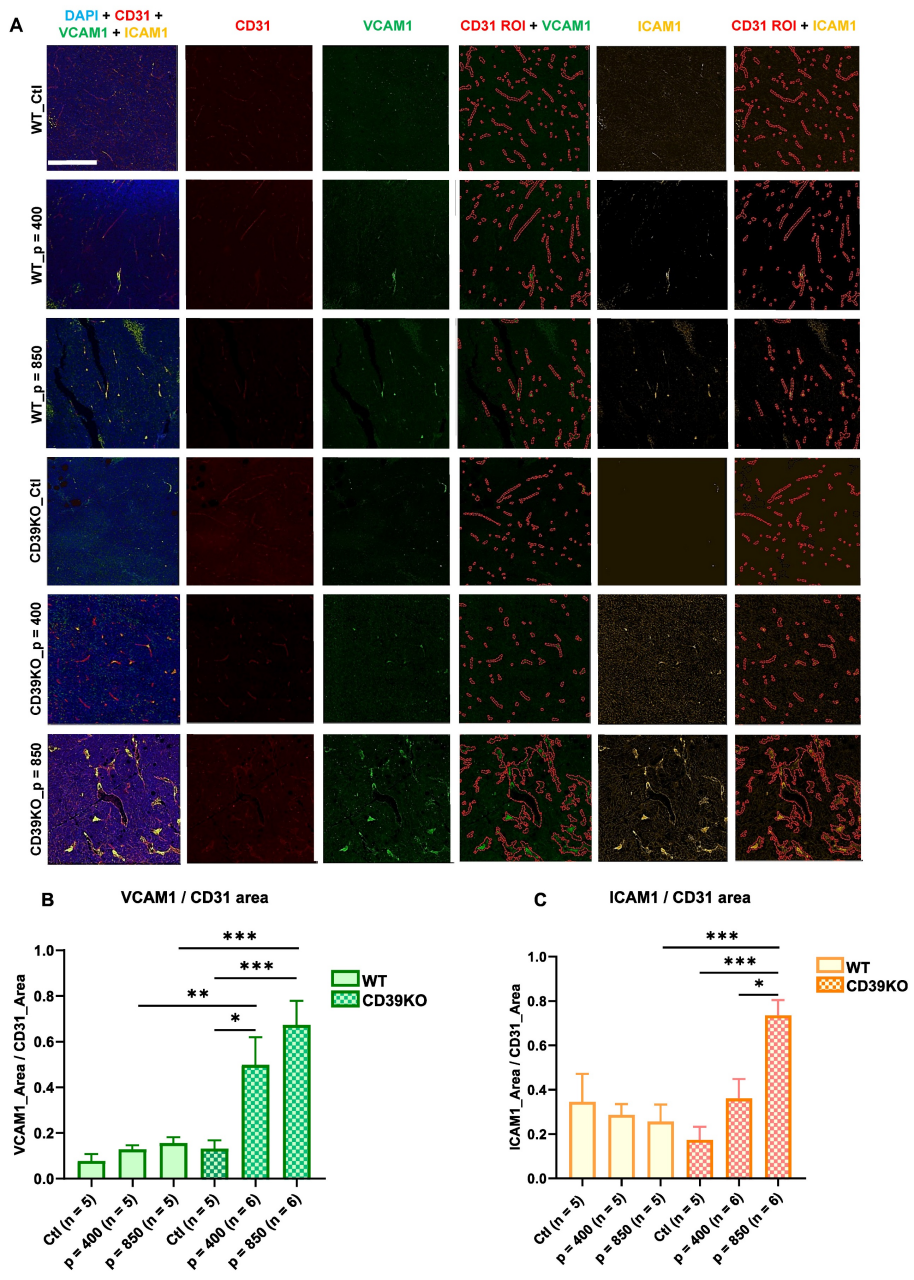
** Vascular inflammation markers following UTMC 24 h post-treatment. A)** Typical multiplex immunofluorescence staining of CD31 (vascular endothelial marker, in red), VCAM-1 (green) and ICAM-1 (yellow) (vascular inflammatory markers). VCAM-1 and ICAM-1 are quantified within CD31 ROIs. The scale bar is 200 µm. **B)** VCAM-1 significantly increased in CD39KO_p = 850 vs. CD39KO_Ctl and WT_p = 850 (p = 0.0004 and 0.0008, resp.). Similarly, VCAM1 expression was higher in CD39KO_p = 400 vs. CD39KO_Ctl and WT_p = 400 (p = 0.02 and 0.009, resp.). **C)** ICAM1 expression in CD39KO_p = 850 was enhanced compared to CD39KO_p = 400 (p = 0.02), CD39KO_Ctl (p = 0.0006) and WT_p = 850 (p = 0.0003). CD39KO: CD39 knock out mice; CD39KO_Ctl: control group of CD39KO mice; ROI: region of interest; US: ultrasound; UTMC: ultrasound targeted microbubble cavitation; WT_Ctl; control group of wild-type mice.

To identify inflammatory responses, the expression of endothelial ICAM-1 and VCAM-1 was assessed using histological analysis on tumors samples at 24 h (**Figure 5**). Immunofluorescence staining against CD31 was used to define the vasculature map, referred to as CD31 ROI. Subsequently, ICAM-1 and VCAM-1 expression within CD31 ROI was quantified. Our results showed that endothelial VCAM-1 expression significantly increased in CD39KO_p = 850 vs. CD39KO_Ctl and WT_p = 850 (p = 0.0004 and 0.0008, resp., **Figure 5B**). Similarly, VCAM1 expression was higher in CD39KO_p = 400 vs. CD39KO_Ctl and WT_p = 400 (p = 0.02 and 0.009, resp.). However, this increase was not observed in WT group. Additionally, endothelial ICAM-1 increased in CD39KO mice treated at 850 kPa compared to p = 400 (p = 0.02) and control group (p = 0.0006;** Figure 5C**). The difference between WT and CD39KO mice for ICAM-1 was significant only at p = 850 kPa UTMC (p = 0.0003).

### UTMC induces cancer cell death and reduces tumor proliferation in WT and CD39KO mice

**Figure 6:**
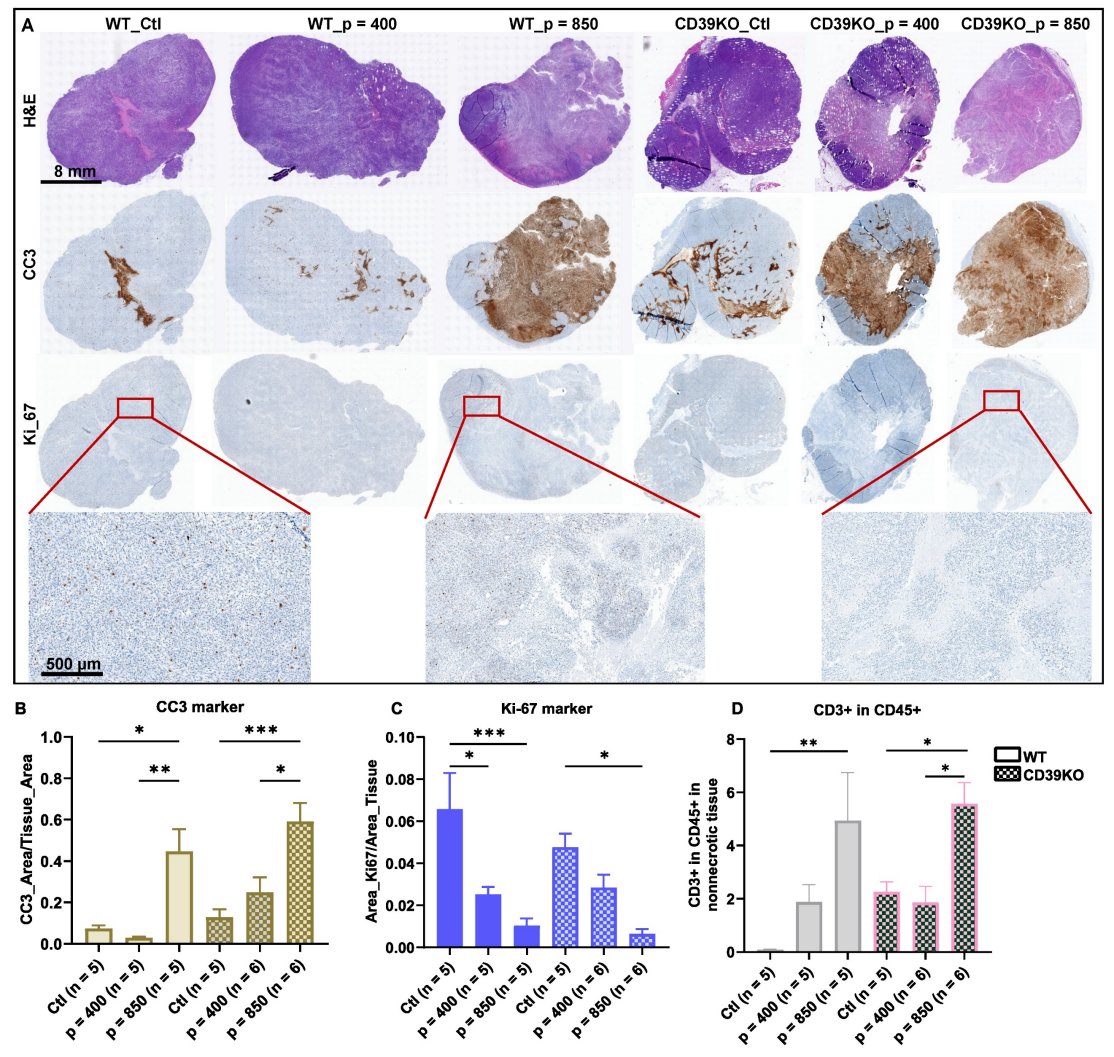
** Cancer cell death, cancer cell proliferation, and T cell infiltration following UTMC at 24 h. A)** Typical H&E, CC3, and Ki-67 staining on sequential slides (scale bar = 8 mm). **B)** CC3 was highest in CD39KO_p = 850 and significantly increased compared to CD39KO_Ctl and CD39KO_p = 400 (p = 0.004 and 0.04, resp.). Differences in CC3 expression were not significant between CD39KO and WT at any UTMC pressure (p = 0.8, 0.4, 0.08, resp.). **C)** Ki-67 was lowest in the CD39KO_p = 850 and it was significantly lower than CD39KO_Ctl (p = 0.003). In the WT group, Ki-67 expression reduced at both US pressure 400 and 850 kPa (p = 0.01 and < 0.0001, resp.). Ki-67 expression was not different between WT vs. CD39KO mice at any UTMC pressure (p = 0.8, 0.4, 0.08, resp.). **D)** The ratio of CD3+ in CD45+ was significantly enhanced in WT_p = 850 compared to WT_Ctl (p = 0.005) and in CD39KO_p = 850 compared to CD39KO_Ctl and CD39KO_p = 400 (p = 0.05 and p = 0.01, resp.). Differences in the ratio of CD3+ in CD45+ was not significant between CD39KO and WT groups at any UTMC pressure (p = 0.19, 0.99, 0.93, resp.). H&E: hematoxylin and eosin; CC3: cleaved caspase 3; Ki-67: antigen Kiel 67; CD39KO_Ctl: control group of CD39 knock out mice; WT_Ctl: control group of wild-type mice.

To investigate the downstream effect of ATP enhancement following UTMC treatment, we studied cancer cell death and proliferation in WT and CD39KO mice with US pressures of 400 and 850 kPa in MC38 tumors at 24 h (**Figure 6A**). IHC staining revealed a significant elevation in CC3 expression in both WT and CD39KO mice following UTMC treatment at 850 kPa compared to the control and 400 kPa groups (**Figure 6B**). While CC3 expression was higher in CD39KO versus WT mice (p = 0.02, two-way ANOVA), post-hoc multiple comparisons did not reveal significant differences at any US pressure. A notable reduction in Ki-67 expression was observed in WT mice following UTMC treatment at both 850 and 400 kPa compared to the control group (p < 0.0001 and 0.01, resp., **Figure 6C**). A similar pattern was detected in CD39KO mice, but significant only when treated with UTMC at 850 kPa (p = 0.003). Ki-67 expression was not significantly different in CD39KO mice compared to WT mice at any US pressure. Finally, IHC staining against the CD3 and CD45 markers was used to detect infiltrated T cells in tumor specimens. Our results showed that 24 h after UTMC, the ratio of CD3+ in CD45+ was significantly enhanced in WT mice at 850 kPa compared to the control group (p = 0.005; **Figure 6D**). In CD39KO mice the ratio of CD3+ in CD45+ cell was significantly increased in CD39KO_p = 850 compared to CD39KO_Ctl (p = 0.05) and CD39KO_p = 400 groups (p = 0.01). The ratio of CD3+ amongst CD45+ infiltrated immune cells was not significantly higher in CD39KO mice compared to WT mice with/without UTMC treatment (p = 0.15, two-way ANOVA).

### Combination therapy of UTMC + aPDL1 + POM-1 reduced tumor growth better than monotherapies

**Figure 7:**
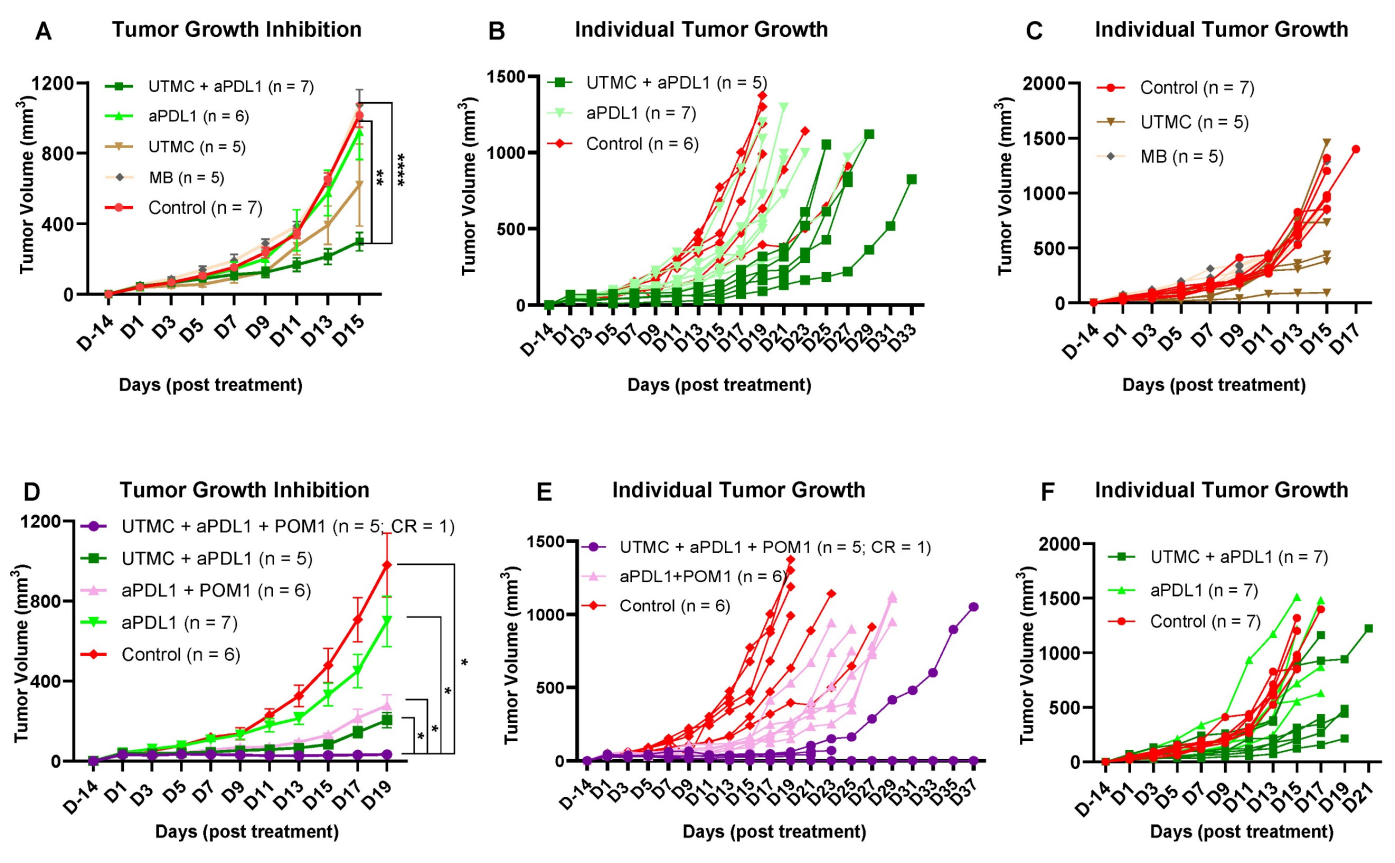
** Tumor growth inhibition following UTMC treatment with a low dose of aPDL1. A)** Tumor growth inhibition following the combination or monotherapy of UTMC (850 kPa), MB and aPDL1 (100 µg). **B & C, E & F)** Individual tumor growth curve for each treatment. **D)** The greatest tumor growth inhibition effect was seen in the fully combined treatment group of UTMC + aPDL1 (100 µg) + POM1 (250 µg) compared to every other treatment. The data present means ± SEM (*P < 0.05, **P < 0.01, ****P < 0.0001, 2-way ANOVA test, multiple comparison test of Sidak). aPDL1: anti-programmed cell death ligand-1; POM-1: Sodium polyoxotungstate-1; UTMC: ultrasound targeted microbubble cavitation; WT: wild-type mice.

Based on the previous acute findings, we selected 850kPa for the rest of the studies. We tested whether UTMC in combination with CD39 inhibition would improve aPDL1 efficacy in WT mice. Our data supported that combining UTMC with a low dose of aPDL1 (100 µg) and CD39 inhibition (250 µg POM-1) can synergize and effectively inhibit tumor growth. Our results indicate that neither UTMC nor aPDL1 monotherapy was sufficient to inhibit tumor growth. However, the combination significantly inhibited tumor growth compared to the control, aPDL1 alone, and MB without US (p < 0.0001, p = 0.017, 0.006, and 0.0008, resp.) (**Figure 7A-C**). This experiment was repeated to observe the effect of CD39 inhibition (**Figure 7D-F**). The combination of UTMC + aPDL1 + POM-1 resulted in the greatest tumor growth inhibition, starting as early as Day 3, compared to control and aPDL1 groups (**Figure 7D** and **Table 1**). One mouse in the UTMC + aPDL1 + POM1 completely rejected the tumor and did not develop tumor in the re-challenge experiment. This study indicates that ATP release following UTMC, in combination with pharmacological CD39 inhibition, improves the efficacy of aPDL1 at a low dose of 100 µg. Additionally, the combination of UTMC and aPDL1 improved mice survival compared to the aPDL1 and control groups (p = 0.03 and p = 0.04, resp., Mantel-Cox log-rank test, **Figure S1**). Three mice in the UTMC + aPDL1 + POM1 group developed clinical complications on Day 21-23 post-treatment. These complications included a 15-20% body weight loss, abnormal gait, and an enlarged gallbladder, likely due to the lack of food intake and dehydration, and were humanely euthanized upon observation. The combined treatment was further tested with a higher dosage of aPDL1 (200 μg), however we lost the improvement in the efficacy of the combination treatment, possibly because aPDL1 (200 μg) monotherapy was very efficacious in these models (**Figure S2-S3**).

### UTMC-mediated eATP enhancement induced tumor infiltration of immune cells

**Figure 8:**
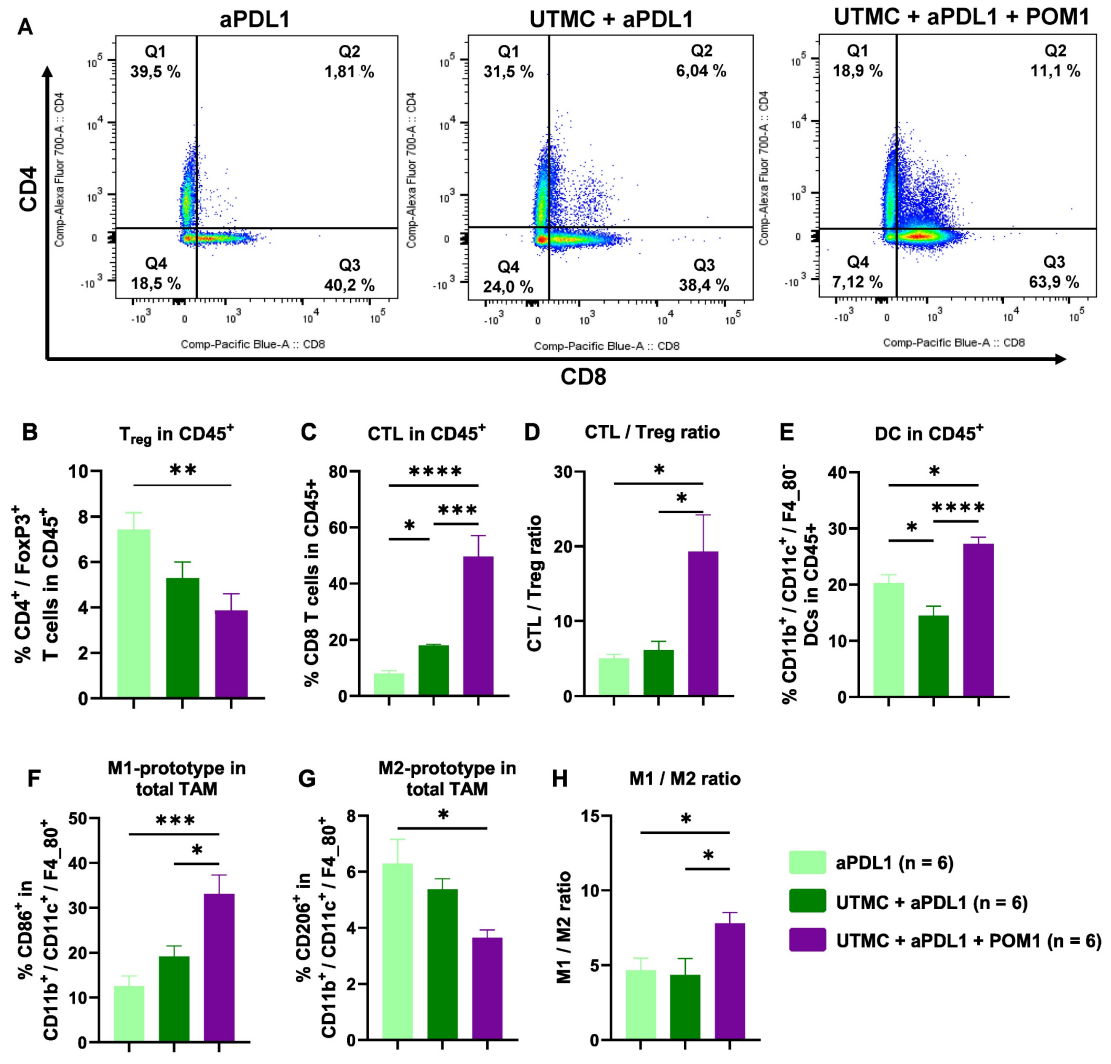
** eATP enhancement following UTMC in combination with aPDL1 induced anti-tumor immune responses. A)** Flow cytometry analysis of the percentage of tumor infiltrated CTLs (Q3 represents CD8+ T cells) and CD4+ T cells in UTMC + aPDL1 + POM1 (n = 6), UTMC + aPDL1 (n = 6), and aPDL1 (n = 8) groups three days after last treatment. **B)** The fully combined treatment (UTMC + aPDL1 + POM1) reduced Tregs compared to aPDL1 monotherapy (p = 0.01). **C)** The fully combined treatment also showed a significant increase in the percentage of CTLs compared to UTMC + aPDL1 (p = 0.009) and aPDL1 (p = 0.001). **D)** The CTL/Treg ratio was enhanced in the fully combined treatment group compared to UTMC + aPDL1 (p = 0.02) and aPDL1 (p = 0.012). **E)** The percentage of DCs was increased in the fully combined treatment compared to UTMC + aPDL1 (p < 0.0001) and aPDL1 (p = 0.011). **F)** M1-prototype TAMs were increased in the fully combined treatment group compared to UTMC + aPDL1 (p = 0.015) and aPDL1 (p = 0.0008). **G)** Conversely, M2-prototype TAMs, typically associated with immunosuppression, were reduced in the fully combined treatment compared to aPDL1 (p = 0.02). **H)** The ratio of M1 to M2 prototype TAMs was significantly increased in the fully combined treatment compared to UTMC + aPDL1 (p = 0.04) and aPDL1 (p = 0.048). aPDL1: anti-programmed cell death ligand-1; CTL: cytotoxic T cells; DC: dendritic cells; POM-1: Sodium polyoxotungstate-1; TAM: tumor associated macrophages; UTMC: ultrasound targeted microbubble cavitation.

Flow cytometry analysis at D11 revealed that UTMC-mediated eATP enhancement shifted the immune cell response towards an inflamed state, with the most pronounced immune cell infiltration observed in the fully combined treatment (UTMC + aPDL1 + POM1) compared to UTMC + aPDL1 or aPDL1 monotherapy (**Figure 8**). The fully combined treatment group exhibited a lower percentage of immunosuppressive Tregs (CD4+/FoxP3+ T cells) versus aPDL1 monotherapy. Notably, this group showed a higher percentage of CTLs (CD8+ T cells) (**Figure 8A-C**), along with an increased CTL/Treg ratio (**Figure 8D**) compared to the other groups, indicating a shift toward a more cytotoxic TME.

Moreover, the fully combined treatment group showed significant changes in the myeloid-derived immune cell subpopulations, with increased DCs and M1-prototype tumor-associated macrophages (TAMs), both associated with pro-inflammatory and anti-tumor activities. In contrast, the percentage of M2-prototype TAMs, which are typically linked to immunosuppression and tumor promotion, was reduced. (**Figure 8G**). Accordingly, the M2/M1 ratio was also reduced with the combined treatment (**Figure 8H**).

### UTMC-mediated eATP enhancement increased immune cell trafficking to the TDLNs

**Figure 9:**
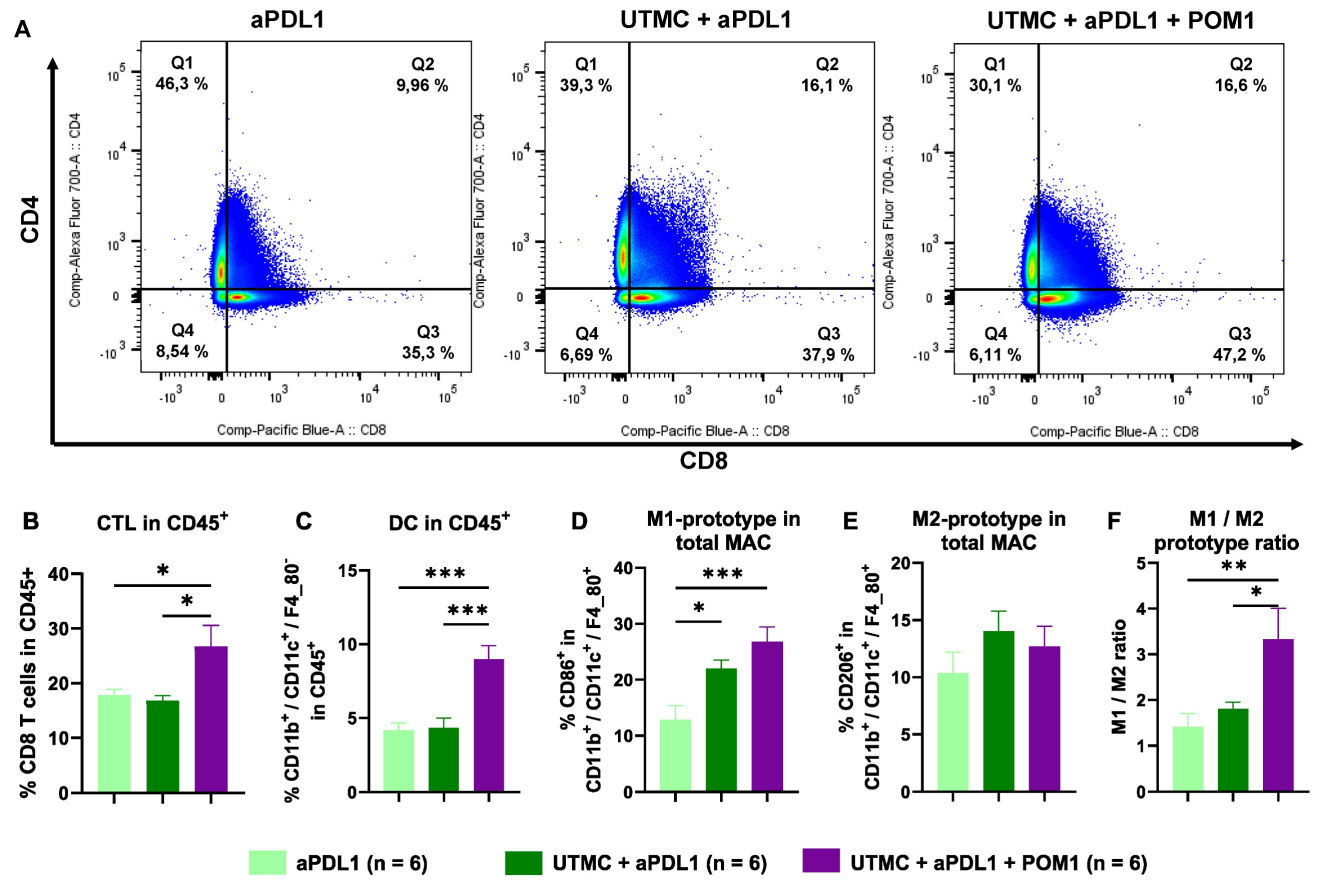
** eATP enhancement following UTMC in combination with aPDL1 increased immune cell trafficking to the TDLNs. A)** Flow cytometry analysis of the percentage of CTLs (Q3 represents CD8+ T cells) and CD4+ T cells in TDLNs of UTMC + aPDL1 + POM1 (n = 7), UTMC + aPDL1 (n = 8), and aPDL1 (n = 8) groups three days after last treatment. **B)** The fully combined treatment increased the percentage of CTLs compared to UTMC + aPDL1 (p = 0.005) and aPDL1 (p = 0.013). **C)** The percentage of DCs was increased in the fully combined treatment group compared to UTMC + aPDL1 (p = 0.0004) and aPDL1 (p = 0.0003). **D)** M1-prototype macrophages were increased in the fully combined treatment group compared to UTMC + aPDL1 (p = 0.001) and aPDL1 (p = 0.046). **E)** However, M2-prototype macrophages did not show any differences across different groups. **F)** The ratio of M1 to M2 prototype TAMs was significantly increased in the fully combined treatment compared to UTMC + aPDL1 (p = 0.03) and aPDL1 (p = 0.008). aPDL1: anti-programmed cell death ligand-1; CTL: cytotoxic T cells; DC: dendritic cells; POM-1: Sodium polyoxotungstate-1; UTMC: ultrasound targeted microbubble cavitation.

Flow cytometry analysis of cells in the TDLNs highlight a higher percentage of CTLs following the full combination treatment compared to UTMC + aPDL1 (p = 0.005) and aPDL1 (p = 0.013) (**Figure 9A-B**). Interestingly, the percentage of DCs increased in UTMC + aPDL1 + POM1 vs. UTMC + aPDL1 (p = 0.004) and aPDL1 monotherapy (p = 0.0003) (**Figure 9C**). M1 macrophages also increased in the UTMC + aPDL1 + POM1 and UTMC + aPDL1 compared to aPDL1 (p = 0.001 and p = 0.046, resp.) (**Figure 9D**). We found no changes in M2 macrophages (**Figure 9E**), but did find an increase in the M1/M2 ratio in UTMC + aPDL1 + POM1 group compared to UTMC + aPDL1 (p = 0.03) and aPDL1 (p = 0.008) (**Figure 9F**).

## Discussion

Understanding and exploiting purinergic signaling and ATP in immune checkpoint blockade therapy is an active area of research in immuno-oncology 12, 14, 15. We recently identified that UTMC could release ATP locally in tumors 45 and postulated that this could be leveraged to improve ICB efficacy in combination with CD39 inhibition. In this study, in an MC38 colorectal carcinoma mouse model, we used non-ablative UTMC at two different US pressures 400 or 850 kPa to enhance tumoral ATP release, provoke immune responses, and improve aPDL1 therapeutic efficacy. Our pre and post UTMC perfusion cineloops support that our UTMC conditions did not cause vascular shutdown **(Figure 2B)** at either tested pressure. This is consistent with other studies exploring the combination of non-ablative UTMC with ICB treatment in pre-clinical models 36, 43, 46. Note that UTMC can be adjusted to either shut down or preserve tumoral perfusion. Both ablative 47-49 and non-ablative 36, 46 UTMC have been shown to stimulate anti-tumor immune responses, thus turning cold TME into hot TME 5, 25, 36. However, it remains unclear which regime (ablative vs non-ablative) is better, and if our results are applicable to ablative UTMC. Histologically, we then examined tissue samples collected 2 h post-UTMC treatment and observed notable vasodilation in all UTMC-treated, with a more pronounced effect at US pressure of 850 kPa in both WT and CD39KO models **(Figure 3)**, compared to untreated controls. We observed enlarged blood vessels, at 2 h post UTMC, a histological confirmation of the ultrasound perfusion data, and suggesting an improvement in tumor vascularization, as also reported by others 36. We compared ATP release in WT and CD39KO mice using bioluminescence imaging. In the CD39KO model, we found that UTMC with p = 850 kPa enhanced eATP release in MC38 tumors compared to the contralateral untreated control, and compared to WT, and remained higher at least for 90 minutes post-UTMC (**Figure 4**), supporting that CD39 inhibition could increase UTMC mediated ATP release. Since ATP is a pro-inflammatory molecule, we investigated downstream vascular inflammation markers, tumor cell viability and immune cell infiltration at 24 h after UTMC treatment using histological techniques. Interestingly, we observed that upon UTMC-mediated ATP release, vascular inflammation markers (ICAM-1/VCAM-1) were upregulated (**Figure 5**) with an increased response in the CD39KO model. Our data consistently showed increased cancer cell death (CC3 expression), and decreased cell proliferation (Ki-67) with increasing UTMC pressure (**Figure 6**).

In aggregate, our acute data supports that ATP released by UTMC causes vascular inflammation and helps recruit T-cells: since immune cells rely on vascular markers like ICAM-1 and VCAM-1 for adhesion and diapedesis, our findings support that vascular inflammation post-UTMC is a mechanism explaining the increased ratio of CD3+ cells found in tumors at 24 h. This finding is in line with previous reports describing UTMC-induced anti-tumor immune responses through the release of DAMPs 36, 43, 50. Recently, UTMC was reported to induce HMGB1 33, 43 and HSP70 expression and calreticulin translocation in MC38 tumors, indicating DAMPs' involvement in recruiting adaptive immune responses 43. However, despite increased ATP and vascular inflammation in CD39KO mice, acute cell death (CC3) and cell proliferation (Ki-67) were not different in the CD39KO model compared to the WT at 24 h (Figure 6B). These findings are consistent with the increased ratio of CD3+ in CD45+ cells infiltrated in tumors treated with UTMC at p = 850 kPa (**Figure 6**) but without a significant effect with CD39 inhibition in the acute response.

The limited effectiveness of ICB therapies against solid tumors has drawn attention to the need for combining other therapeutic modalities with ICBs, including UTMC 5, 51, 52. In this study, we combined ICB with non-ablative UTMC to maintain tumor permeability and promote an immune-stimulatory environment, thereby potentially improving the efficacy of aPDL1 in the MC38 model. Our longitudinal studies demonstrate that MB alone, or UTMC alone did not cause growth inhibition in our model (**Figure 7A-C**), supporting that MB toxicity or possible vascular damage was not a major driver in our UTMC conditions. Yet, with a 100 µg aPDL1 dose (a dose insufficient in monotherapy) we found a greater tumor growth inhibition following UTMC + aPDL1, consistent with several other studies that combined ICB with UTMC 36, 43, 46. One mechanism often reported is an increase in mAb extravasation with UTMC, as described by us and others 36, 42, 46, 53. Most interestingly, combining UTMC + aPDL1 + POM1 dramatically inhibited MC38 tumor growth (**Figures 7D-F**), and was significantly better than all other treatment combinations, including UTMC + aPDL1. Interestingly, aPDL1 + POM1 was also better than aPDL1 alone, as expected 11, which supports that UTMC can further improve the pharmacological inhibition of CD39, likely by increasing eATP. In the UTMC + aPDL1 + POM1 group, one mouse completely rejected the tumor, and upon re-challenge, it did not develop a tumor for up to 70 days.

Not surprisingly, we found that this synergistic effect was dose-dependent: in the supplementary material, we show that with an increased dose of aPDL1 (200 µg), we lost the improvement in efficacy of the combination treatment, possibly because monotherapy was very efficacious in this model (**Figure S2-S3**).

To further investigate the mechanisms of the treatment efficacy, we studied the cellular immune response in the tumors and TDLN at day 11. Flow cytometry results of tumoral immune cell infiltration indicate that UTMC-mediated eATP enhancement combined with POM1 and aPDL1 (100 µg), significantly shifted the immune landscape within the TME toward an inflamed state (**Figure 8**). The UTMC + aPDL1 + POM1 showed an enhanced tumoral immune cell infiltration compared to either UTMC + aPDL1 or aPDL1 monotherapy, with a higher percentage of CTLs and a reduced percentage of immunosuppressive Tregs. The increased CTL/Treg ratio highlights a more favorable immune environment for tumor clearance. Additionally, the combination treatment elevated pro-inflammatory myeloid-derived immune cell subpopulations, including DCs and M1-prototype TAMs, while reducing the immunosuppressive M2-prototype TAMs, all of which promote enhanced antigen presentation, immune activation, and immunogenic cancer cell death. Our results align with previous studies that underscore the importance of modulating the TME to improve ICB efficacy. For instance, Li et al. (2019) demonstrated that CD39 blockade is fully dependent on host lymphocytes, enhancing intratumoral T-cell activation and converting a cold, non-inflamed TME into a hot, inflamed one, thus making it more responsive to aPDL1 therapy 11. Also, Li et al. (2021) showed that UTMC improved CTL infiltration and facilitated aPDL1 delivery 36. Tang et al. (2023) further emphasized that ultrasound-based therapies depend on CTL-mediated immunity and DC-driven antigen presentation, which is consistent with our finding of increased DCs and M1-TAMs, essential for initiating CTL-driven anti-tumor immune responses 43. Our findings are in line with those studies but additionally support the important role of ATP and CD39 inhibition in promoting immune reprogramming.

To further investigate the implication of the adaptive immune system in the anti-tumor response, we also analyzed the TDLNs. In TDLNs, the UTMC + aPDL1 + POM1 treatment also increased CTLs, DCs, and M1-prototype macrophages suggesting heightened immune activation, and antigen presentation (**Figure 9**), which indicated a possible immune activation at the systemic level. The higher M1/M2 macrophage ratio further underscores the anti-tumor effects of this combination treatment. TDLNs play a crucial role in DC-mediated antigen presentation to CTLs, thereby orchestrating and amplifying anti-tumor immune responses.

Altogether, our longitudinal data supports that a local UTMC treatment on the tumor, combined with CD39 inhibition, elicits a strong adaptive immune response that we could detect in the tumor and in the tumor draining lymph nodes. This response could dramatically improve the response to a low dose aPDL1 in our rodent model. Interestingly, our data were also consistent with previous findings, as the combination of CD39 blockade and aPDL1 11 as well as UTMC and aPDL1 36, 39, has been shown to enhance CTL infiltration and improve aPDL1-mediated anti-tumor immune responses. However, our results demonstrate even greater immune activation following the combination of UTMC, aPDL1, and the CD39 inhibitor (POM1). In addition to T cells, our data also supports the activation of myeloid-derived immune subsets in the combination therapy.

The safety of UTMC as a spatially targeted therapy has been largely demonstrated most notably in the brain where it is being clinically tested for BBB 42. At the preclinical level, many groups have combined focused ultrasound and immunotherapy successfully and safely 36, 46, 47, 54, 55. Nevertheless, the safety of UTMC and focused ultrasound in general depends on the ultrasound parameters used and the precise localisation of the ultrasound energy. Compared to HIFU or histotripsy or even ablative UTMC, our mild flow preserving US parameters are not expected to cause safety concerns. In terms of combining UTMC with ICB and CD39 inhibitions, the safety of the approach remains to be fully established.

Our data strongly supports that CD39 inhibition can increase ATP release by UTMC and improve the immune response in this model. Please note that we used a genetic knock out model in our acute studies and a small molecule systemic inhibitor in our longitudinal studies. The reason for this was that we could not measure bioluminescence in the presence of POM1. Please note that in the CD39KO model, we found an elevated ATP signal at baseline, which was expected. For the efficacy studies, the use of a pharmacological approach served three purposes: (1) it allowed a more accurate comparison between groups all composed of WT mice; (2) it allowed to demonstrate that CD39 is targetable with a small molecule; (3) it reduced chronic effects of CD39 knock down. The full physiological effects of CD39 inhibitions, which are beyond the scope of this paper, can be found here 56-58. Further investigation is needed to elucidate the interactions between different cell types in the immune response and the respective roles of innate and adaptive immunity. The strength of the adaptive immune stimulation also remains to be studied in an abscopal model.

## Conclusion

In conclusion, this study demonstrates that non-ablative UTMC increased extracellular ATP in our subcutaneous tumor model, particularly for CD39KO mice. Vascular inflammation, which also increased with UTMC and CD39 inhibition, is proposed as a mechanism induced by tumoral ATP release, contributing to anti-tumor immune responses. At 24 h post-treatment, UTMC at p = 850 kPa induced cancer cell death and reduced cancer cell proliferation, which could indicate an innate immune response induced by ATP at this time point. Most interestingly, the combination of UTMC + aPDL1 + POM1 caused dramatic MC38 tumor growth inhibition compared to all other combination therapies. At day 11, this treatment also increased cytotoxic T cells (CTL), the CTL/Treg ratio, dendritic cells, and M1-prototype tumor-associated macrophages, while reducing M2-prototype macrophages within the tumor. In the TDLNs, the fully combined treatment elevated CTLs, dendritic cells, and M1-prototype macrophages, with a concurrent reduction in M2-prototype macrophages. To the best of our knowledge, this is the first study to demonstrate the role of ATP in UTMC-mediated enhanced ICB efficacy, which paves the way for leveraging purinergic signaling in immune check point inhibition using ultrasound targeted image guided therapy.

## Supplementary Material

Supplementary figures.

## Figures and Tables

**Table 1:** Statistical differences at different time points between treatment groups in Figure 7D

Groups	aPDL1 + POM1			UTMC + aPDL1			UTMC + aPDL1 + POM1		
Dates	D3	D7	D19	D3	D7	D19	D3	D7	D19
Control		0.008			0.005	0.02	0.0021	0.001	0.01
aPDL1	0.04	0.005	0.03		0.008	0.03	0.0002	0.0005	0.01
aPDL1 + POM1									0.02
UTMC + aPDL1									0.04

## Abbreviations

aPDL1: anti-programmed cell death ligand-1
BLI: bioluminescent imaging
CD3: cluster of differentiation 3
CD45: cluster of differentiation 45
CD39KO: CD39 knock out mice
CEUS: contrast enhanced ultrasonography
CTL: cytotoxic T cells
DAMP: danger associated molecular pattern
DC: dendritic cells
eATP: extracellular ATP
FUS: focused ultrasound
H&E: hematoxylin and eosin staining
HIFU: high intensity focused ultrasound
ICAM-1: intercellular adhesion molecule-1
IHC: immunohistochemistry
IF: immunofluorescent
MAC: macrophage MB: microbubble
MI: mechanical index
POM-1: sodium polyoxotungstate
ROI: region of Interest
TAM: tumor associated macrophage
TDLN: tumor-draining lymph node
TME: tumor microenvironment
Treg: regulatory T cells
UTMC: ultrasound targeted microbubble cavitation
VCAM-1: vascular cell adhesion molecule 1
WT: wild type mice
