# Supplementary material for: ATP released by ultrasound targeted microbubble cavitation induces vascular inflammation and improves immune checkpoint blockade efficacy
Source: Theranostics. 2025 Apr 11;15(11):5220–37. doi: 10.7150/thno.105857 (PMC12036883; doi:10.7150/thno.105857)
Supplement: Supplementary file 1 — Supplementary figures. [file thnov15p5220s1.pdf]

Supporting Information

Additional experimental results

ATP released by Ultrasound Targeted Microbubble Cavitation Induces vascular inflammation and Antitumor Inflammatory Responses Improves Immune Checkpoint Blockade Efficacy

Sepideh Jahangiri [1,2,3], Samuel Bourdages [1,2,4], Emma Skora[2,5], John Stagg [2,5] and François Yu[1,2,4,6]*

[1]Microbubble Theranostics Laboratory, CHUM Research Center, Montreal, Canada
[2]Institut du Cancer de Montréal, Montreal, Canada
[3]Faculty of Medicine, Université de Montréal, Montreal, Canada
[4]Biomedical Engineering Institute, Université de Montréal, Montreal, Canada
[5]Faculty of Pharmacy, Université de Montréal, Montreal, Canada.
[6]Department of Radiology, Radiation Oncology and Nuclear Medicine, Faculty of Medicine, Université de Montréal, Montreal, Canada

* Corresponding author: francois.yu@umontreal.ca

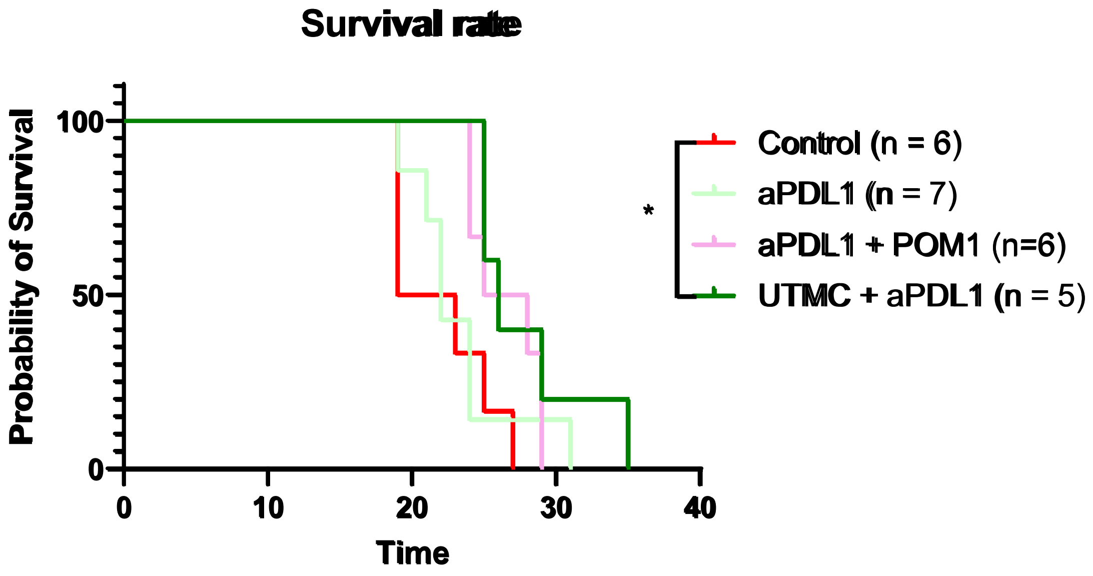

Figure S1. Combined UTMC and aPDL1 (100 µg) improved mice survival in a WT mice model of MC38. Improved mice survival following the combined treatment of UTMC + aPDL1 versus aPDL1 monotherapy and the control groups ($p = 0.03$ and $p = 0.04$, resp., Mantel-Cox log-rank test). Although, one mouse in the UTMC + aPDL1 + POM1 group completely rejected the tumor, the survival data for this group is not included in this figure because the mice were euthanized due to clinical complications unrelated to tumor progression, which appeared on D21-23. Clinical complications in three out of five mice in UTMC + aPDL1 + POM1 group included 15 - 20% body weight loss, abnormal gait, and an enlarged gallbladder due to lack of food intake and dehydration. An independent veterinarian conducted the evaluation clarified that the symptoms were unlikely to be linked to the treatment or anesthesia cytotoxicity, as such effects would typically appear within 24 - 48 h after treatment.

aPDL1: anti-programmed cell death ligand-1; POM-1: Sodium polyoxotungstate-1; s.c.: subcutaneous; UTMC: ultrasound targeted microbubble cavitation.

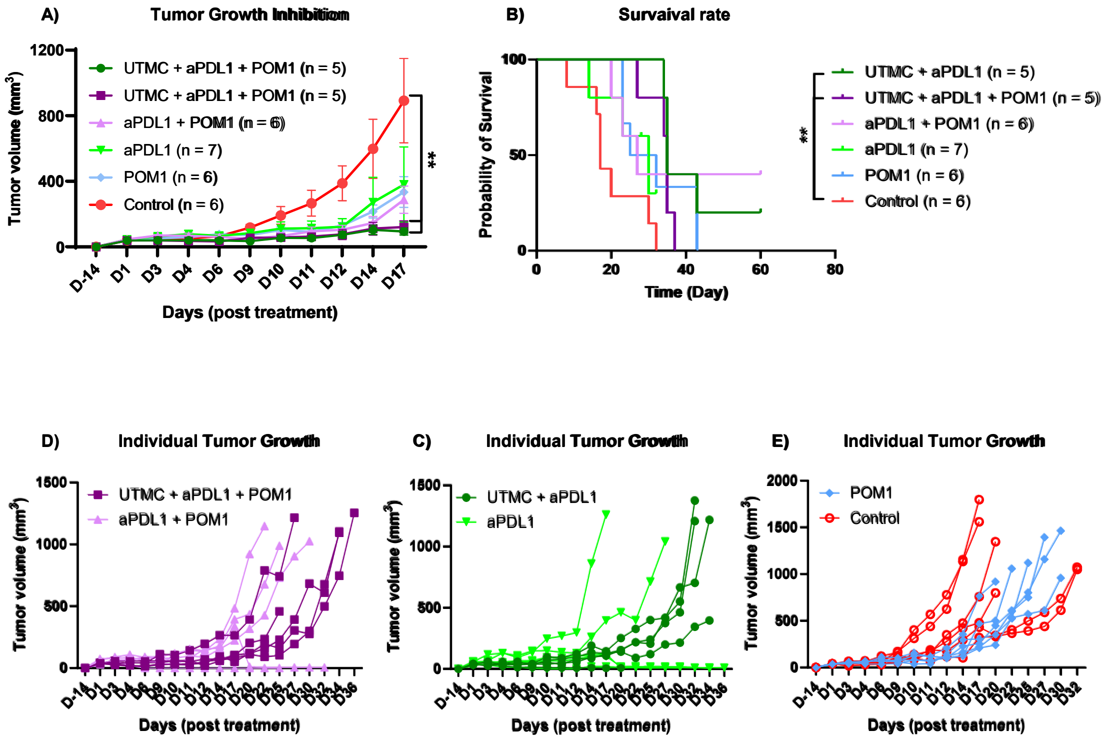

Figure S2. Tumor inhibition of combined UTMC and aPDL1 (200 µg) treatment in subcutaneous WT model of MC38. A) MC38 tumor-bearing WT mice were treated with UTMC (p = 850 kPa), aPDL1 antibody (200 µg) and POM-1 (250 µg) every 2-3 days, beginning on the average tumor size of 40 mm^3 (n > 5 per group) for 3 times. By day 17, the UTMC + aPDL1 and UTMC + aPDL1 + POM1 treated mice had a 6.5 and 5- fold reduction in tumor size compared with the control group, respectively. Tumor growth in UTMC + aPDL1 + POM1 and UTMC + aPDL1 was significantly inhibited compared to the control group ($p = 0.0047$ and 0.0087, resp., Mann-Whitney test). The tumor sizes were not significantly different in UTMC + aPDL1 + POM1 vs UTMC + aPD-L1 group at any point before D17. Mice with complete rejection of tumor (CR) were entered to the re-challenge experiment by receiving a second s.c. injection of MC38 ($2.5*10^6$) on the contralateral flank. No tumor development was observed in any CR mice during two months follow-up period. **B)** Kaplan-Meier survival curves for each treatment group represent a stronger increase in mice survival in both UTMC + aPDL1 + POM1 and UTMC + aPDL1 groups ($p = 0.008$ and 0.001, respectively). **C-E)** Tumor growth curve was measured every 2-3 days. Mice were humanly sacrificed when they reached the cut-off size of 1000 mm^3. The data presents mean ± SEM.

aPDL1: anti-programmed cell death ligand-1; POM-1: Sodium polyoxotungstate-1; UTMC: ultrasound targeted microbubble cavitation; WT; wild-type mice.

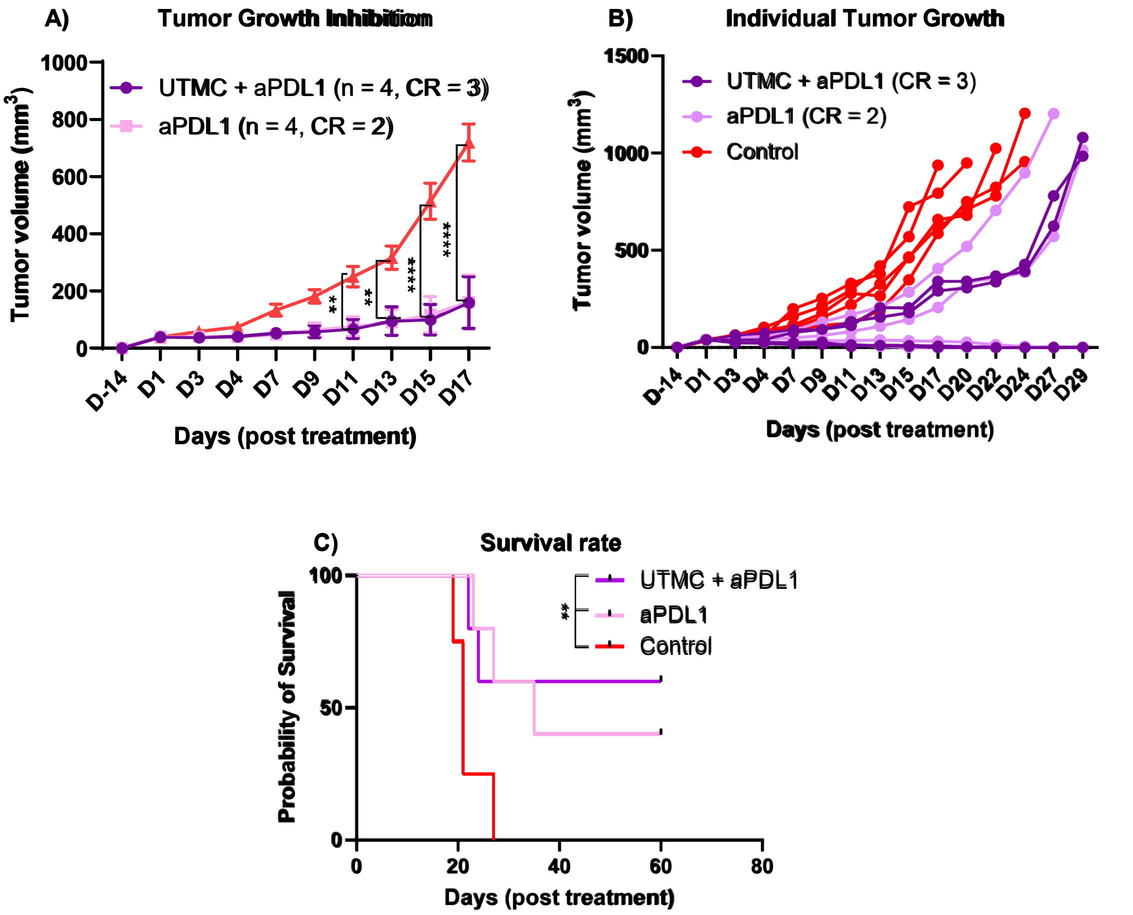

Figure S3. Efficacy study of UTMC + aPDL1 (200 µg) in CD39KO mice. A) UTMC + aPDL1 and aPDL1 reduced tumor growth in CD39KO mice. Data is represented as mean $\pm$ SEM. **B)** Individual tumor growth curves. **C)** The Kaplan-Meier survival curve indicates improved survival in both UTMC + aPDL1 and aPDL1 groups. Mice with complete rejection of tumor (CR) entered to the re-challenge experiment by receiving a second s.c. injection of MC38 ($2.5*10^6$) on the contralateral flank. No tumor development was observed in any CR mice during two months follow-up period.

aPDL1: anti-programmed cell death ligand-1; CD39KO: CD39 knock out mice; UTMC: ultrasound targeted microbubble cavitation.
